# Draft genome sequence of multidrug-resistant *Citrobacter portucalensis* BAU_133-2 strain isolated from a domestic duck (*Anas platyrhynchos domesticus*) in Bangladesh

**DOI:** 10.1128/MRA.00587-23

**Published:** 2023-11-01

**Authors:** Tarana Ahmed, Md. Saiful Islam, Mohammad Nuruzzaman, Mohammad Sadekuzzaman, S. M. Lutful Kabir, Md. Tanvir Rahman, Md. Shahidur Rahman Khan

**Affiliations:** 1 Department of Microbiology and Hygiene, Bangladesh Agricultural University, Mymensingh, Bangladesh; 2 Department of Livestock Services, Ministry of Fisheries & Livestock, Government of the Peoples Republic of Bangladesh, Krishi Khamar Sarak, Farmgate, Dhaka, Bangladesh; 3 Department of Animal Sciences, University of California—Davis, Davis, California, USA; 4 Ministry of Public Administration, Dhaka, Bangladesh; 5 Department of Livestock Services, Central Disease Investigation Laboratory (CDIL), Ministry of Fisheries & Livestock, Government of the Peoples Republic of Bangladesh, Krishi Khamar Sarak, Farmgate, Dhaka, Bangladesh; University of Maryland School of Medicine, Baltimore, Maryland, USA

**Keywords:** *Citrobacter portucalensis*, duck, WGS, antimicrobial resistance, virulence determinants, metabolic functional features, Bangladesh

## Abstract

We announce the genome sequence of the *Citrobacter portucalensis* BAU_133-2 strain isolated from a domestic duck. Our assembled genome contained a length of 4.8 Mb, 110.0× genome coverage, 51.91% of an average GC content, 1 plasmid, 1 CRISPR array, 8 prophages, 27 antibiotic resistance genes, and 75 virulence factor genes.

## ANNOUNCEMENT

The widespread and improper utilization of antimicrobial agents has led to the rise of antimicrobial resistance in bacteria, giving rise to numerous multidrug-resistant (MDR) strains, which have now become a significant worldwide public health issue (1, 2). Ducks possess the capability to carry antimicrobial-resistant and MDR pathogens that may be transmitted to humans through their interactions with them (1, 3). *C. portucalensis* shows the potential to emerge as a critically important bacteria of global significance for both public health and One Health (4). Previously, antimicrobial-resistant *C. portucalensis* was isolated from humans, animals, and environments throughout the globe (5–8).

All the protocols and methodologies associated with the current study were approved by the Bangladesh Agricultural University’s ethics committee, “Animal Welfare and Experimentation Ethical Committee” [AWEEC/BAU/2020(10)], Mymensingh, Bangladesh. Between January 2020 and January 2022, cloacal swab samples of diseased wild domestic ducks (*Anas platyrhynchos domesticus*) were taken from the Kishoreganj District (24.4260° N, 90.9821° E) of Bangladesh and transported to the laboratory. These samples were incubated overnight in nutrient broth (HiMedia, India) at 37°C. The incubated samples were streaked on xylose-lysine deoxycholate agar (HiMedia, India) media, and the resulting pure colonies underwent staining and biochemical tests to isolate *C. portucalensis* (9). The MDR properties of the isolates were ascertained through the disk diffusion method (10) and the CLSI guidelines (11). The *C. portucalensis* BAU_133-2 isolate, showing phenotypic resistance to gentamicin, ciprofloxacin, cephalexin, azithromycin, tetracycline, ampicillin, cotrimoxazole, imipenem, and cefotaxime, was selected in this study. This isolate was cultured in nutrient broth (HiMedia, India) at 37°C overnight, and its complete genomic DNA was obtained from the broth culture using the Qiagen DNA mini kit (QIAGEN, Hilden, Germany). The extracted DNA from the isolate was then subjected to enzymatic fragmentation using the NEBNextdsDNA Fragmentase kit (NEB, MA, USA) according to the manufacturer’s instructions. The fragmented DNA was subjected to size selection using SPRI beads, which helped in isolating DNA fragments of the desired size range for sequencing (12). Subsequently, a sequencing library was generated using the Nextera DNA Flex library prep kit (Illumina, San Diego, CA, USA). The library was then sequenced on the Illumina NextSeq2000 platform using 2  × 150 paired-end reads. The genome assembly was performed using the Unicycler.v0.4.9 (13), and the raw paired-end reads (*n* = 7,587,670) were trimmed using Trimmomatic.v0.39 (14) (with parameters leading:20, slidingwindow:4:20:20, trailing:20, minlen = 36), with the aim of eliminating Illumina adapters, recognized Illumina irregularities, and phiX reads from the data set and the quality was evaluated using FastQC.v0.11.7 (15). Following that, the genome was annotated using PGAP.v3.0 (16). The antibiotic resistance genes (ARGs) were identified using CARD.v3.2.7 with RGI.v6.0.2 (17) and ResFinder.v4.1 (18); virulence factor genes (VFGs) by VFDB with VFanalyzer (19); the sequence type by MLST.v2.0 (20); metabolic functional features from RAST.v2.0 (21); and CRISPR array and prophages by CRISPRimmunity (22) within the assembled genome. Default parameters were used except where otherwise noted.

The draft assembly of the *C. portucalensis* BAU_133-2 genome comprised 36 contigs, a G + C content of 51.91%, 4 contig L50, and the N50 value was 532,725 bp. The genome size of *C. portucalensis* BAU_133-2 was 4,881,935  bp, with a genome coverage of 110.0×. This genome had a total of 4,574 genes, 4,493 CDSs, 71 transfer RNA genes, 4 ribosomal RNA genes, and 58 pseudogenes. This assembled genome identified one CRISPR array (with three signature genes, i.e., *cas3*, *csa3*, and *DEDDh*), eight prophages, and one plasmid [IncFIB(pB171)]. According to MLST analysis, this genome corresponds to sequence type ST211. The strain carried 27 predicted ARGs, 75 predicted VFGs, 383 subsystems with 34% coverage, and 2,118 genes.

## Data Availability

The research on *C. portucalensis* BAU_133-2, conducted using the WGS shotgun approach, was submitted to NCBI/GenBank, and it was assigned the accession number JAPQVZ000000000. The pertinent data, including the original readings, were stored with BioProject accession number PRJNA907481, BioSample accession number SAMN31981961, and SRA accession number SRR24872939. The specific version mentioned in this document is labeled as JAPQVZ000000000.1.
